# Supplementary material for: Seasonal Stability and Dynamics of DNA Methylation in Plants in a Natural Environment
Source: Genes (Basel). 2019 Jul 17;10(7):544. doi: 10.3390/genes10070544 (PMC6679105; doi:10.3390/genes10070544)
Supplement: Supplementary file 1 [file genes-10-00544-s001.pdf]

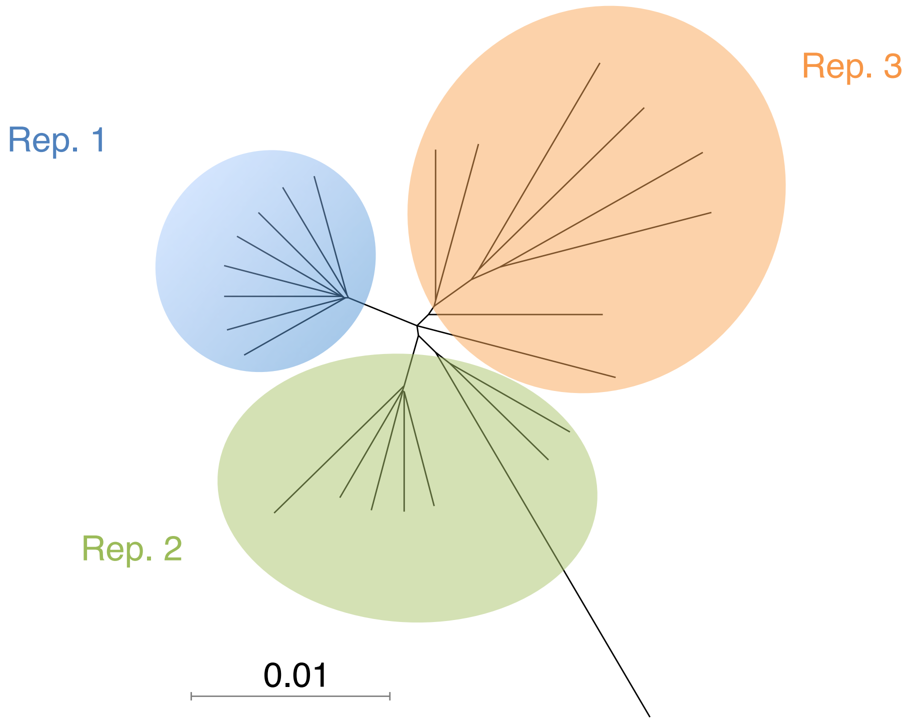

Fig. S1. Some patches of A. halleri turned to be genetically mixed. A dendrogram shows Kimura's genetic distance using genome-wide SNPs among the samples in three patches for eight timepoints. The samples from replicate 1 were judged to be a single clone. A. halleri is an obligate outcrossing species with a self-incompatible breeding system, and therefore a large portion of SNPs are expected to be heterozygous. Because heterozygous SNPs can be designated as homozygous to either of the alleles in a certain probability, a terminal radial branching is expected to be observed even for genetically identical plants that belong to a single clonal patch (Rep. 1).

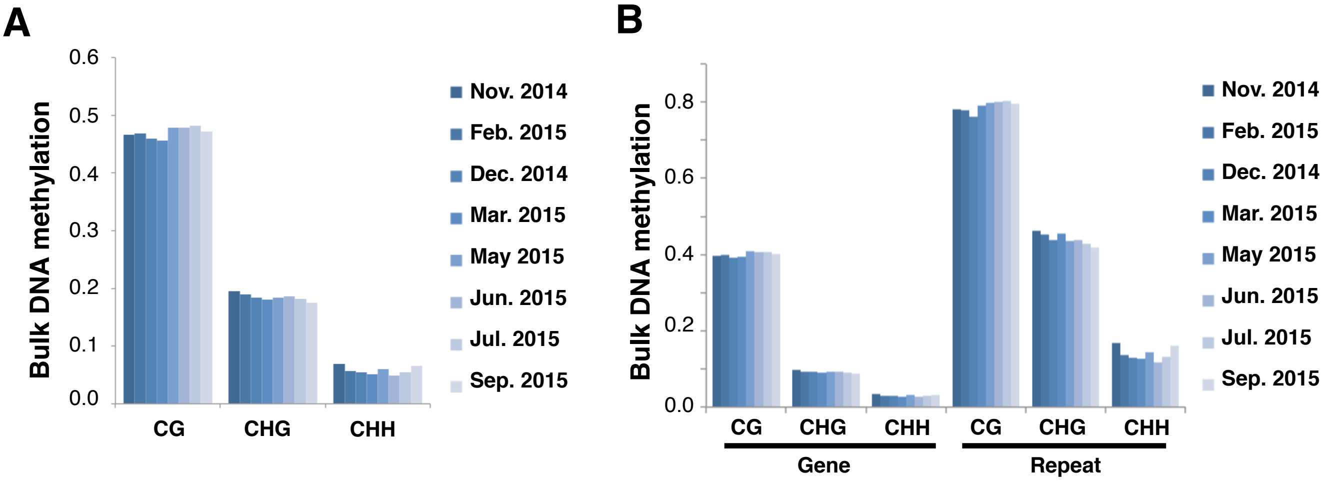

Fig. S2. Genome-wide bulk DNA methylation level at eight time points across a year. Genome-wide bulk DNA methylation levels are shown in CG, CHG, and CHH context for the whole genome (*A*), and gene and repetitive sequences (*B*). Eight sampling timings are represented by the different shades.

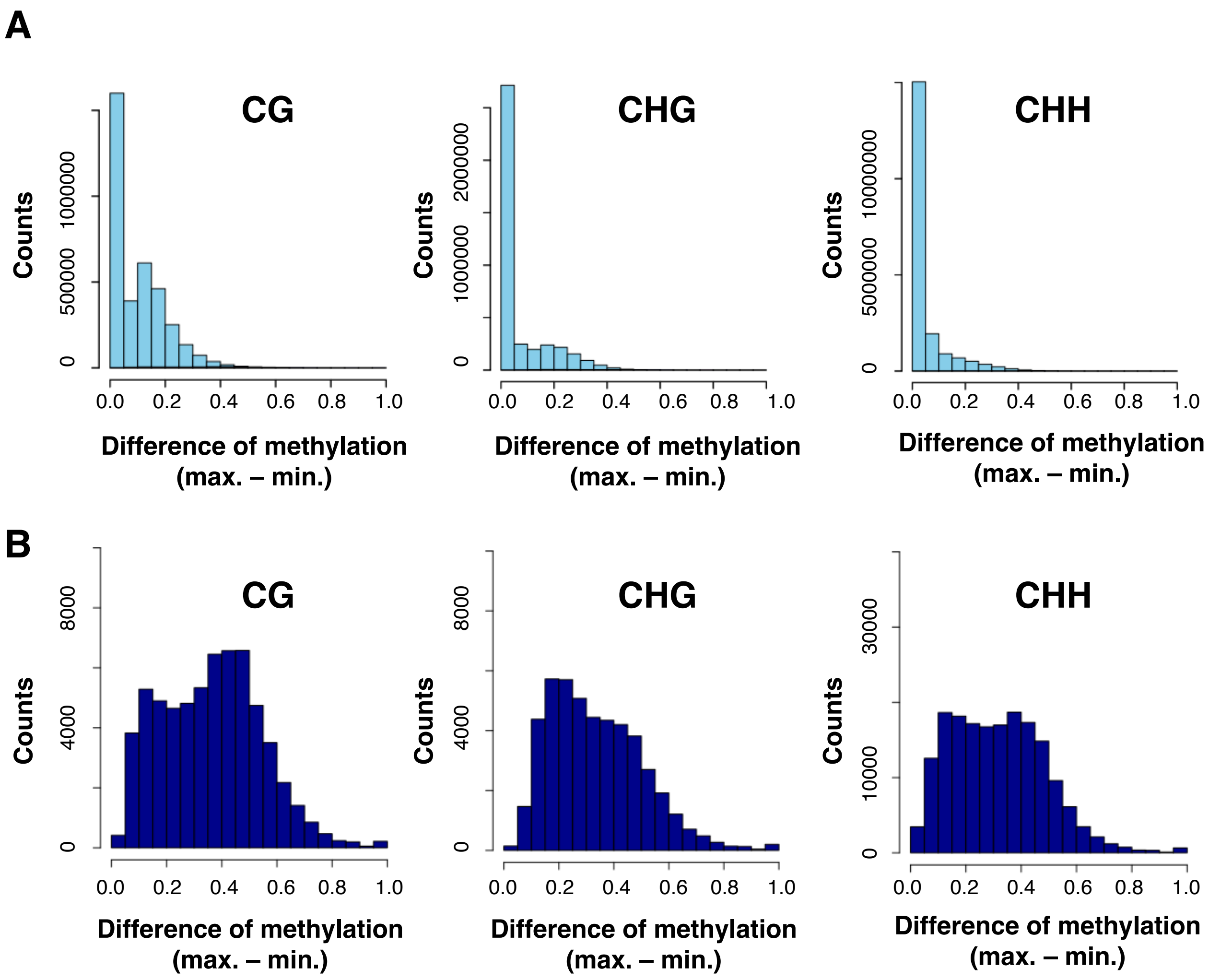

Fig. S3. DNA methylation was seasonally stable at a majority of CG, CHG, and CHH sites. Histograms of seasonal differences of DNA methylation (max. – min.) for all cytosine sites (*A*), and for SeMCs (*B*) in CG, CHG, and CHH contexts.

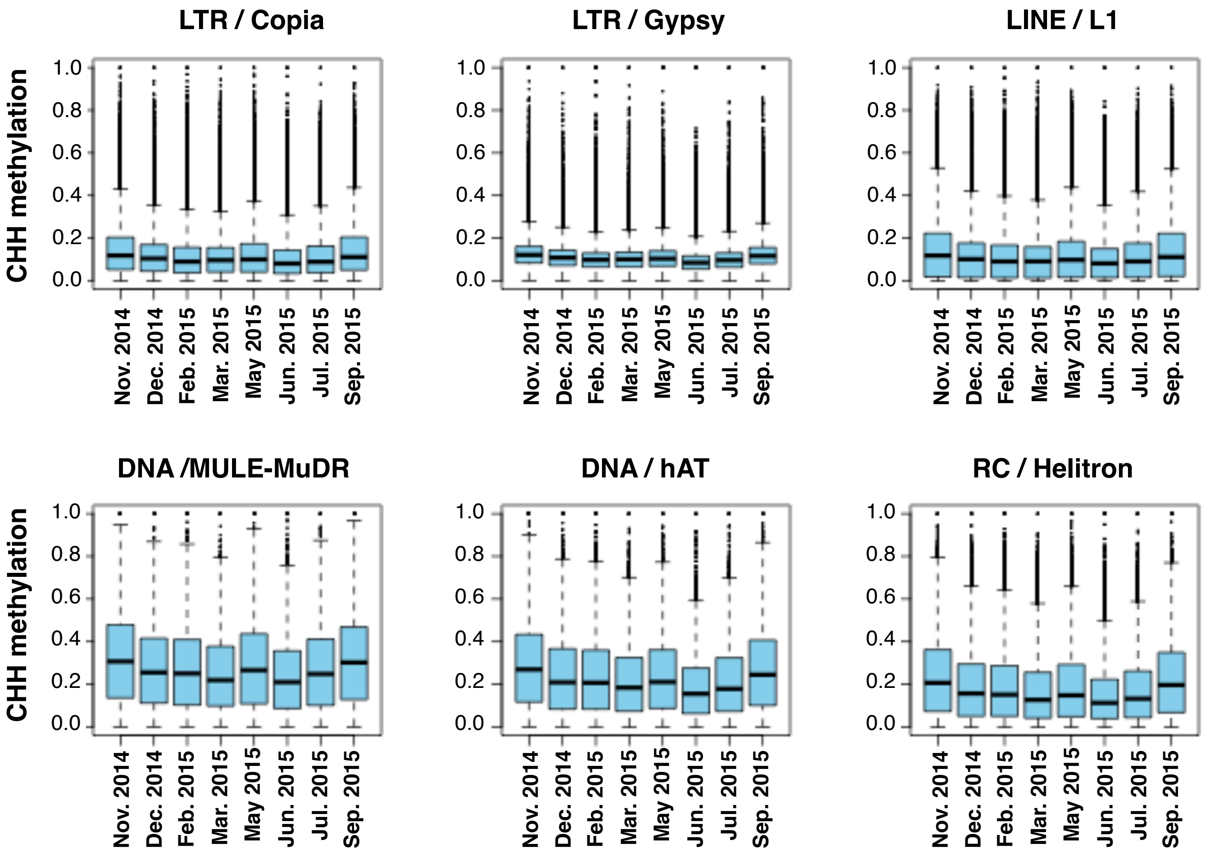

Fig. S4. Seasonal patterns in CHH DNA methylation in repetitive elements that belong to the six major families of transposable elements (TEs). The boxes span from the first to the third quartiles, the thick black bars inside the boxes are the medians, whiskers above and below the boxes represent 1.5 × interquartile ranges from the quartiles.

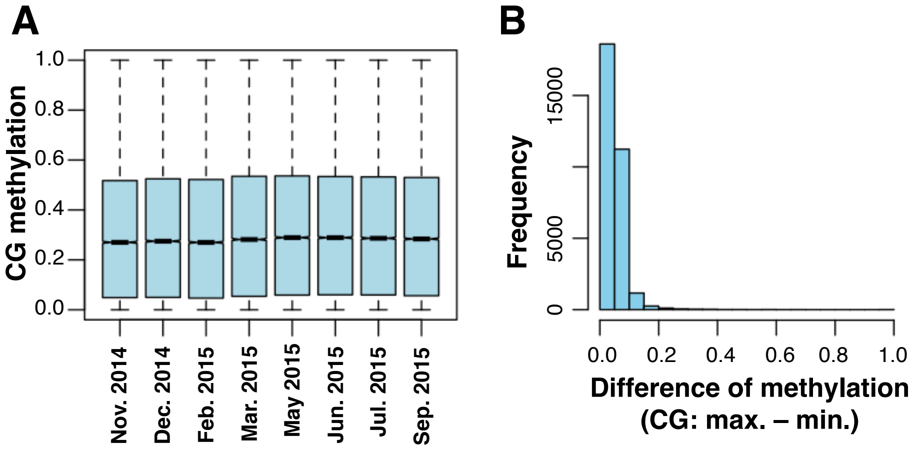

Fig. S5. Seasonal patterns in CG gene body methylation (gbM) across a year. (*A*) Boxplot of DNA methylation in genes in CG context at eight time points across a year. The boxes span from the first to the third quartiles, the thick black bars inside the boxes are the medians, whiskers above and below the boxes represent 1.5 × interquartile ranges from the quartiles. (*B*) A histogram of seasonal difference for DNA methylation in genes (max. – min.) in CG context.

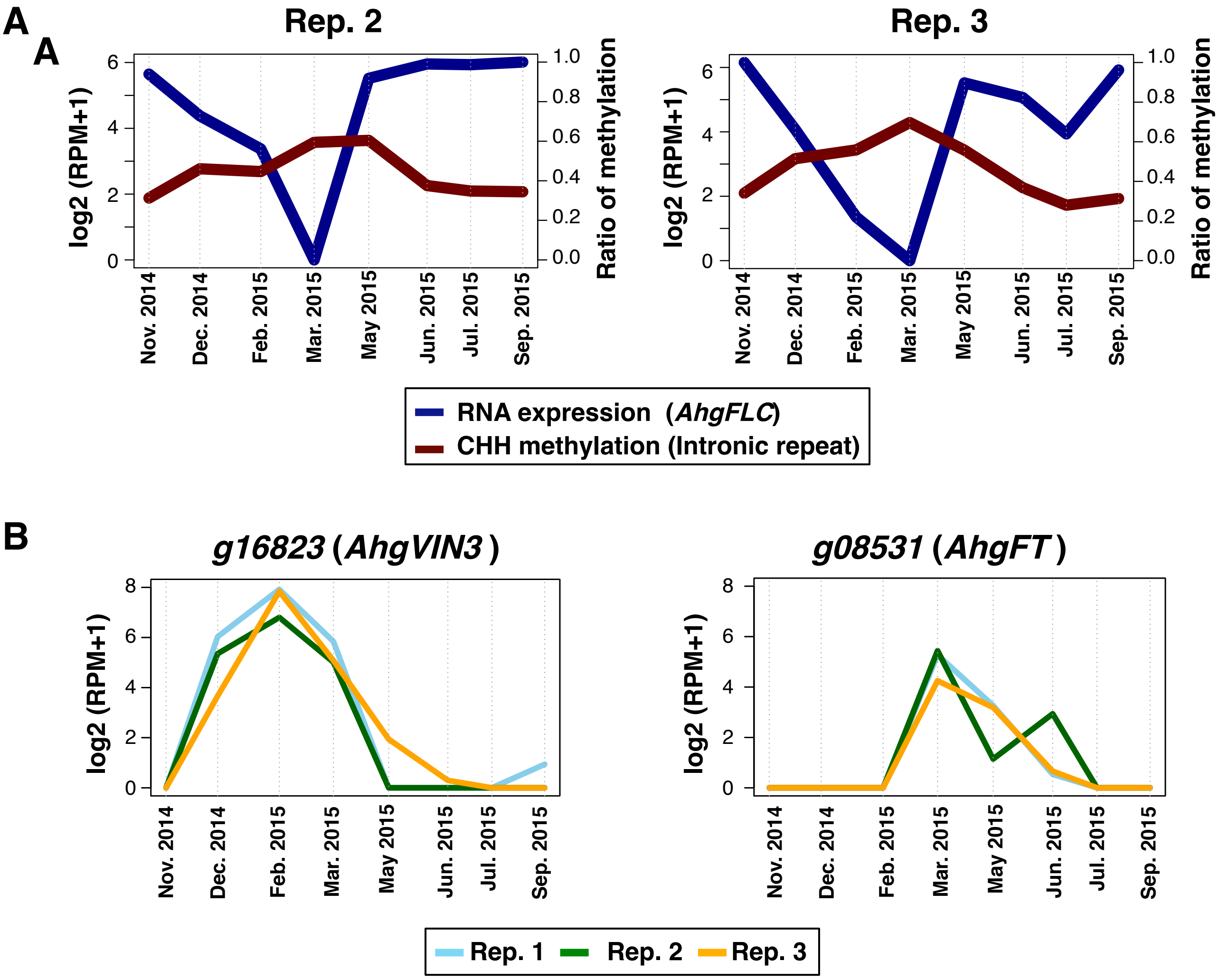

Fig. S6. Seasonal patterns of flowering loci were reproducibly observed. (*A*) Comparison between RNA expression of *AhgFLC* and CHH methylation of the repetitive sequence in its intron, as shown in Fig. 4. The data for Rep. 2 and Rep. 3 are shown. (*B*) Seasonal dynamics of RNA expression in *AhgVIN3* (left) and *AhgFT* (right) is shown. The data for all three patches are shown.

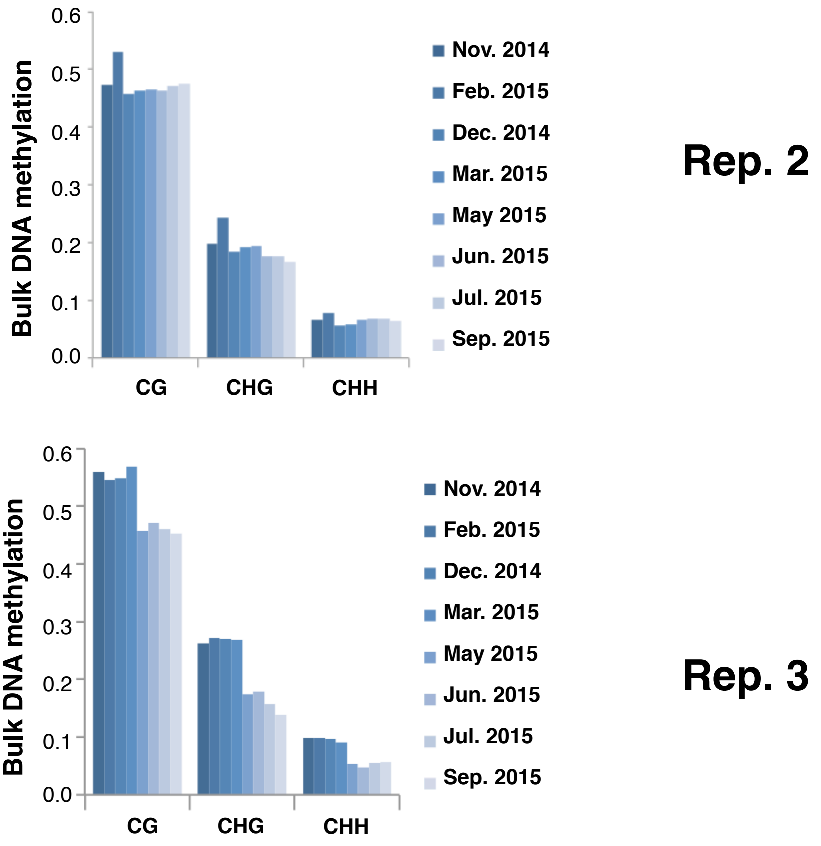

Fig. S7. Genetically mixed patches showed lineage effect on genome-wide DNA methylation. Genome-wide bulk DNA methylation levels are shown in CG, CHG, and CHH context for the whole genome data for Rep. 2 and Rep. 3, as shown in Fig S2A. Eight sampling timings are represented by the different shades.

Table S1. Efficiency of bisulfite treatment in this study.

Sampling time	Rep1	Rep2	Rep3
Nov. 2014	99.34%	99.39%	99.30%
Dec. 2014	99.38%	99.37%	99.41%
Feb. 2015	99.42%	99.34%	99.24%
Mar. 2015	99.39%	99.34%	99.25%
May. 2015	99.29%	99.35%	99.34%
Jun. 2015	99.42%	99.36%	99.38%
Jul. 2015	99.48%	99.30%	99.48%
Sep. 2015	99.46%	99.35%	99.56%

Table S2. Summary of sequencing and mapping for the genome-wide DNA methylation data at 8 sampling times.

Sampling time	Number of processed reads	Number of mapped reads	Mapping efficiency
Nov. 2014	90,520,893	40,941,176	45.20%
Dec. 2014	87,303,234	37,824,643	43.30%
Feb. 2015	92,067,366	38,579,387	41.90%
Mar. 2015	90,115,863	34,016,251	37.70%
May. 2015	91,491,555	38,257,975	41.80%
Jun. 2015	89,141,892	37,461,447	42.00%
Jul. 2015	91,279,854	39,459,871	43.20%
Sep. 2015	89,745,835	40,284,890	44.90%

Sampling time	Number of covered cytosine (depth > 0)	Number of covered cytosine(depth > 4)*	Average depth (depth > 0)*
Nov. 2014	54,230,923	51,825,183	30.33
Dec. 2014	54,204,436	51,694,499	28.7
Feb. 2015	54,182,477	51,706,302	28.36
Mar. 2015	54,086,068	51,368,590	26.02
May. 2015	54,133,573	51,577,158	28.35
Jun. 2015	54,113,633	51,493,982	27.43
Jul. 2015	54,146,050	51,717,448	29.03
Sep. 2015	54,193,102	51,863,604	30.5

Total number of cytosine in both strand in *A. halleri* genome is 60,159,809.
Total length of the reference sequence is 196,243,198 bp.
*The number and the depth were calculated using a merged data set for eight time points; Cytosines sequenced with depth > 0 in all time points were used.

Table S3. Summary of correlation between DNA methylation and density of repeats in 100 kbp windows at 8 sampling times.

	Pearson's Correlation coefficient		
Sampling time	CG	CHG	CHH
Nov. 2014	0.64	0.68	0.70
Dec. 2014	0.63	0.67	0.68
Feb. 2015	0.63	0.68	0.69
Mar. 2015	0.63	0.67	0.68
May. 2015	0.64	0.67	0.68
Jun. 2015	0.64	0.67	0.67
Jul. 2015	0.64	0.68	0.68
Sep. 2015	0.64	0.68	0.70

Table S4. Enrichment of Gene Ontology in the 'group 5' of Fig. 5 E and F.

Ontology: Component

GO term	Description	P-value	FDR q-value
GO:0099568	cytoplasmic region	1.94E-04	9.29E-02
GO:0005938	cell cortex	1.94E-04	4.64E-02
